# Quantum chemical simulation of the silica-anaesthetic, silica-polymer, and polymer-anaesthetic interactions

**DOI:** 10.1186/s11671-015-0820-8

**Published:** 2015-03-12

**Authors:** Victoriya Payentko, Tetyana Kulyk, Volodymyr Kuts

**Affiliations:** Department of Amorphous and Structurally Ordered Oxides, Chuiko Institute of Surface Chemistry, National Academy of Sciences of Ukraine, 17, General Naumov str., Kyiv, 03164 Ukraine; Department of Physico-chemistry of Nanoporous and Nanosized Carbon Materials, Chuiko Institute of Surface Chemistry, National Academy of Sciences of Ukraine, 17, General Naumov str., Kyiv, 03164 Ukraine

**Keywords:** Quantum chemical simulation, Silica, Polymer, Anaesthetic, 31.15.bu, 71.10.-w, 73.20.At, 73.43.Cd

## Abstract

Using semiempirical PM3 method, a comparative quantum chemical estimation has been carried out of the energy of articaine adsorption on the surfaces of the following composite materials: silica-anaesthetic, polymer-anaesthetic, and silica-polymer-anaesthetic. It has been found that adsorption on silica surface takes place due to electrostatic and nonspecific interactions. The data of quantum chemical calculations of the structures of composite materials may be useful in the creation of different forms of medicine preparations with adjustment characteristics.

## Background

Silica-polymer systems, due to their organic-inorganic nature, are perspective for use as immobilized forms of medicine preparations [1-4]. In particular, the inclusion of anaesthetics into such nanocomposites may provide the prolongation of their activity, rise bioaccesibility, and reduce irritating influence on the organism [5].

Model conceptions on silica-anaesthetic, silica-polymer, and polymer-anaesthetic interactions are of great importance when creating application forms of anaesthetics. The developed method of approach will permit the prognosticating of adjusting educe of preparations as dependent on the chemical nature of components of the composite material. Articaine is widely used in anaesthesia practice. Its inclusion into the composition of application preparations will permit a decrease in their toxic influence on the patients with high sensitiveness.

In the present work, a comparative quantum chemical analysis has been performed of the values of adsorption energies in the silica-anaesthetic, polymer-anaesthetic, silica-polymer, and silica-polymer-anaesthetic systems using semi-empirical PM3 method [6,7].

## Methods

The initial coordinates of atoms in the structures under investigation were determined by molecular mechanics method using HyperChem7 program taking into account electrostatic and disperse forces between the cluster host (silica, polymer) and the guest molecule (articaine). Coordinates found by this way were used in further calculation of the electronic structure of nanocomposites by semi-empirical PM3 method by means of MOPAC 2012 14.083 W program which gives an adequate description of both intermolecular interactions and energetics of cluster formation.

**Figure 1:**
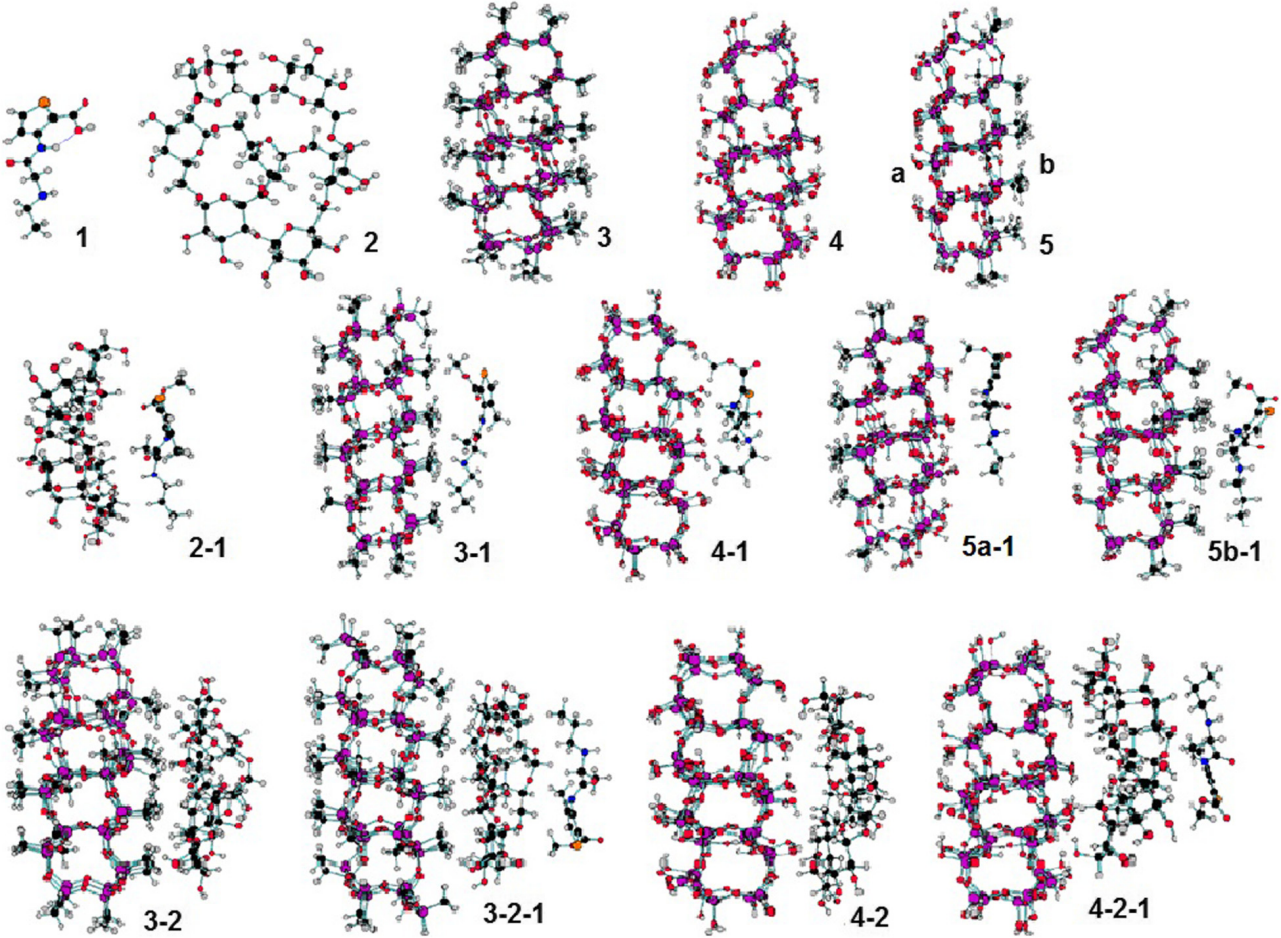
**Model structures under investigation.** Structures of articaine (1); polymer (2); silica nanoclusters (3, 4, 5); binary composites: polymer-articaine (2-1), silica-articaine (3-1, 4-1, 5-1), and silica-polymer (3-2, 4-2); and ternary composites: silica-polymer-articaine (3-2-1, 4-2-1).

Models for silica structure are presented as two planes of nanocluster (SiO_2_)_m_ with different surface nature. In the first case, there are both sides of hydroxylated surfaces of the plane (O_105_Si_50_H_50_); in the second one, the hydroxyls of one side of the plane are changed by methyl groups (nanocluster C_7_O_99_Si_50_H_64_). A model of polymer was simulated by a nanocluster consisting of seven glucose molecules connected to each other with 1.6- of α-1.6 glycosidic linkages between glucose molecules, while branches begin from α-1.3 linkages. Anaesthetic articaine has the formula C_13_N_2_O_3_SH_20_. In silica nanoclusters, the uncompensated valences of edged Si atoms were compensated by two hydrogen atoms (for conservation of sp^3^ hybridization) [8]. Model structures under investigation for articaine (1); polymer (2); silica nanoclusters (3-5); binary composites: polymer-articaine (2-1), silica-articaine (3-1, 4-1, 5-1), and silica-polymer (3-2, 4-2); and ternary composites: silica-polymer-articaine (3-2-1, 4-2-1) are represented in Figure 1.

## Results and discussion

In the present work, the total bonding energy *E*_bd_, the values of adsorption energy *E*_ad_ of articaine on silica and polymer surfaces, and also the charge magnitude on the articaine molecule in binary (polymer-articaine, silica-articaine, silica-polymer) and ternary (silica-polymer-articaine) composite materials have been calculated. Taking into account the use of supramolecular approximation for modelling of adsorption interactions, the values of adsorption energy of articaine on the surfaces under investigation have been determined from the formula [9]:

$$ {E}_{\mathrm{ad}} = E\left(\mathrm{surface}+\mathrm{articaine}\right) - E\left(\mathrm{surface}\right)-E\left(\mathrm{articaine}\right) $$

As it can be seen from Table 1, the values of adsorption energy of articaine molecule *E*_ad_ as dependent on the change in the surface nature become variable in the interval from −22 to −55 kJ/mol that by the order of magnitude of adsorption energy is comparable with the energy of alone hydrogen bond of O-H…O or O-H…N type. The increase of adsorption energy of articaine molecule *E*_ad_ on the silica-dextrane composites (3-2 and 4-2) can be probably ascribed to 2.3 times rise of surface contact for dextrane (727 Ǻ^2^) relatively to articaine (315 Ǻ^2^). It should be noted that the value of adsorption energy of articaine *E*_ad_ on hydroxylated (hydrophilic) surfaces of the silica matrix (4-1, 5a-1, 4-2-1) is always from 2.0 to 4.0 kJ/mol greater than that on the methylated (hydrophobic) surfaces.

**Table 1:** **Model nanoclusters and composite values of** ***E***_**ad**_ **of articaine,** ∆***ρ*** **, and** ***Е***_**HOMO**_ **and** ***Е***_**LUMO**_

**Nanoclusters** ^**a**^ **and composite materials**	***E***_**bd**_ **(eV)**	**∆** ***Е***_**ad**_ **(kJ/mol)**	**∆** ***ρ*** **(** ***e*** **)**	***Е***_**HOMO**_ **(eV)**	***Е***_**LUMO**_ **(eV)**
1	−3,240.84716			−9.265	−1.015
2	−15,685.88357			−10.408	−1.551
3	−44,167.94579			−4.912	4.136
4	−51,616.84388			−5.324	4.351
5	−47,964.94665			−5.557	4.143
2-1	−18,926.95902	−22,029	0.0038		
3-1	−47,409.11363	−30,945	−0.0352		
4-1	−54,858.02645	−323,665	−0.1092		
5а-1	−51,206.36105	−54,737	−0.0510		
5b-1	−51,206.33845	−52,557	−0.0323		
3-2	−59,855.50188	−161,393	−0.1190	−5.122	2.865
4-2	−67,304.03594	−126,265	−0.1366	−5.709	0.133
3-2-1	−63,096.64194	−28,264	−0.0088		

^a^The structures of nanoclusters of composites are given in accordance with Figure 1.

Besides, the extent of charge transfer ∆*ρ* between interacting components for all the composites studied has limits from +0.0038 *е* tо –0.0510 *е*. We suppose that the obtained small values of both adsorption energies *E*_ad_ and extent of charge transfer for silica of different nature and dextrane may be connected with realizing of adsorption processes in such systems mainly due to electrostatic and nonspecific interactions.

As it has been mentioned above, the adsorption of articaine molecule on modified silica surfaces gives changes not only of the extent of charge transfer but also the sign. Really when articaine molecule is adsorbed on dextrane, it becomes positively charged whereas other cases give Δ*ρ* < 0. An analysis of energy values of the frontier molecular orbitals (*E*_HOMO_, the highest occupied molecular orbital, and *E*_LUMO_, the lowest vacant molecular orbital, characterizing respectively electron-donating and electron-accepting properties of the systems) demonstrates that the change of the sign for ∆*ρ* at articaine molecule is defined by the sign of difference Δ*Е* = *Е*_HOMO_(articaine) − *Е*_LUMO_(sorbent) [10].

**Figure 2:**
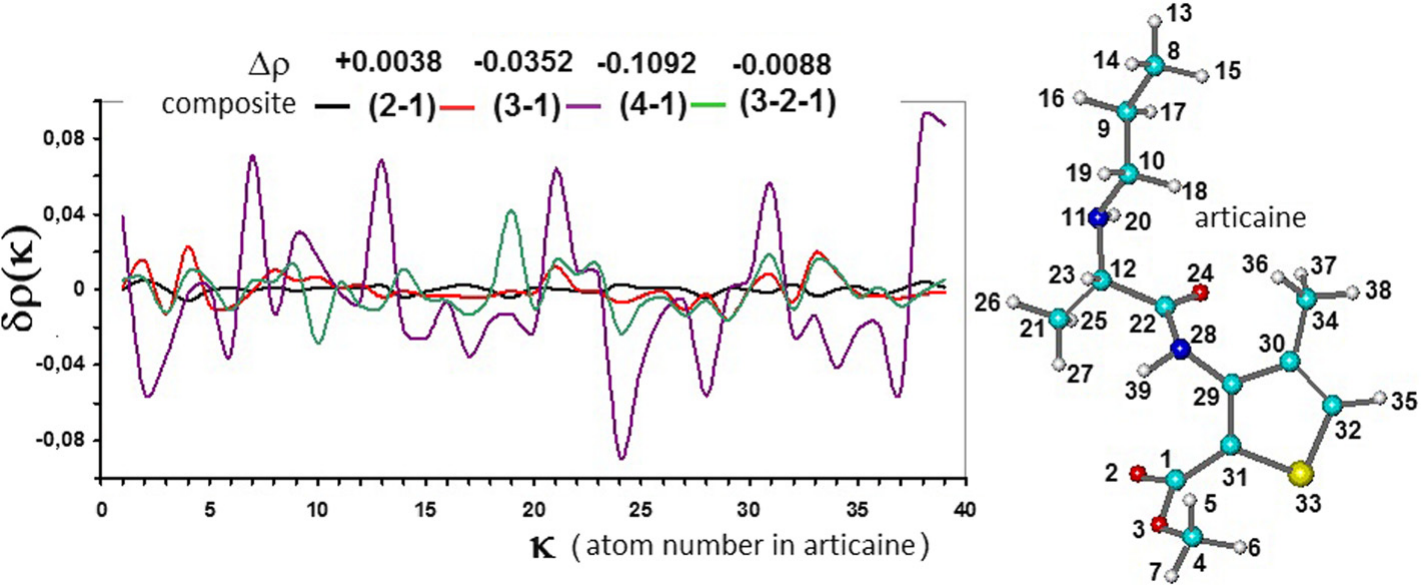
**Dependencies of** ***δρ***_**i**_ **.** For composites dextran-articaine (2-1), methylated silica-articaine (3-1), hydroxylated silica-articaine (4-1), and methylated silica-dextran-articaine (3-2-1).

The observed change of the charge value ∆*ρ* for studied composites gives evidence about the change of electron state of adsorbed molecule. Analysing change of differential electronic density on atoms of the adsorbed molecule in relation to that of not sorbed one *δρ*_i_ = Δ*ρ*_i_^ad^ − Δ*ρ*_i_, it is possible to define the positions of the atoms the most perturbed due to adsorption. Dependencies of *δρ*_i_ for composites dextran-articaine (2-1), methylated silica-articaine (3-1), hydroxylated silica-articaine (4-1), and methylated silica-dextran-articaine (3-2-1) are given as an example in Figure 2.

The absolute changes in *δρ*_i_ on atoms of articaine (Figure 2) in both adsorbed and non-adsorbed states depend on the nature of sorbent surface, but short contacts with the surfaces of all the composites studied are formed mainly by such articaine atoms: carbonyl-type oxygen atom О_2_; methyl groups С_4_Н_3_, С_21_Н_3_, and С_34_Н_3_; propyl group С8-С10; and atoms of thiophene ring.

## Conclusions

It has been found that adsorption in silica surface takes place due to electrostatic interactions. For articaine adsorption on hydroxylated silica surface, the energy value is 1.5 to 4.0 kJ/mol higher than that on methylated silica. An analysis of the values of adsorption energy for articaine in binary (‘dextran-articaine’ and ‘silica-articaine’) and ternary (‘silica-dextran-articaine’) composites shows that changing the nature of the sorbent surface it is probable to affect on the adsorption energy. The results of quantum chemical calculations of the structures of composite materials may be useful in the creation of different forms of medicine preparations with adjustment characteristics.

## Abbreviations

*E*_ad_: Adsorption energy
*E*_bd_: Total bonding energy
*E*_HOMO_: Energy of the highest occupied molecular orbital
*E*_LUMO_: Energy of the lowest vacant molecular orbital
∆*ρ*: Values of total net charge on an articaine molecule on the surfaces of composites
∆*ρ*_i_: Net charge on i-atom of articaine molecule
*δρ*_i_: Differential charge value on i-atom of articaine molecule
